# METTL3 facilitates the translation of CircSIK2 during chicken myogenesis in an m^6^A dependent manner

**DOI:** 10.1371/journal.pgen.1011934

**Published:** 2025-10-31

**Authors:** Zhijun Wang, Xing Ju, Shiru Li, Jiaao Yu, Xin Yang, Danfeng Cai, Kan Li, John M. Gonzalez, Qinghua Nie, Zhenhui Li

**Affiliations:** 1 State Key Laboratory of Swine and Poultry Breeding Industry, Guangdong Laboratory of Lingnan Modern Agriculture, South China Agricultural University, Guangzhou, China; 2 Guangdong Provincial Key Lab of Agro-Animal Genomics and Molecular Breeding, Key Laboratory of Chicken Genetics, Breeding and Reproduction, Ministry of Agriculture, and College of Animal Science, South China Agricultural University, Guangzhou, China; 3 College of Animal Science and Technology, Zhejiang Agriculture and Forestry University, Lin’an, China; 4 Department of Animal and Dairy Sciences, University of Georgia, Athens, Georgia, United States of America; University of California San Francisco, UNITED STATES OF AMERICA

## Abstract

Circular RNAs (circRNAs) are generally considered a new class of non-coding RNA (ncRNA) that frequently appears in the eukaryotic transcriptome. In principle, circRNAs may encode proteins, as some of them are generated from exons and possess elements for internal ribosome entry. Circular RNAs have the potential to serve as an unexplored reservoir for the generation of novel proteins, yet the identification of coding-circRNAs is a daunting task. In this study, we developed a specialized strategy for the discovery of coding-circRNA by combining RNA sequencing, ribosome profiling, and mass spectrometry to find a multitude of circRNAs translated *in vivo*. A total of 40,084 circRNAs were found in chicken myoblasts and myotubes, and 15,332 circRNAs had a predicted open reading frame (ORF). Via ribosome footprints, we discovered that a group of circRNAs (4,069) was associated with translating ribosomes (ribo-circRNAs). Moreover, a total of 3,927 circRNAs with an infinite ORF were discovered, and 860 of them were associated with translating ribosome (ribo-no-stop-codon circRNAs). Mass spectrometry found 5 specific peptides spectra spanning a back-splice junction of circRNAs. circSIK2, one of the ribo-circRNAs, could be methylated by METTL3 and translated into SIK2-176aa, thus promoting the proliferation and differentiation of myoblasts and muscle hypertrophy. Our results suggest that many circRNAs were translating during chicken myogenesis, and METTL3 could enhance the translation of circSIK2. To the best of our knowledge, only two circRNAs translation events have been reported to be mediated by m^6^A. Our research would represent the third such event, and the first documented instance of a translatable circRNA in poultry.

## Author summary

The majority of circular RNAs (circRNAs) originate from exons, typically harboring a complete open reading frame and residing within the cytoplasm. Concurrently, owing to their minimal base sequence divergence from their parental genes, these circRNAs often share a substantial number of m^6^A modification sites with their parent genes. Consequently, unlike the previously defined non-coding RNAs, these circRNAs theoretically possess considerable translation potential. Given the inadequacy of existing primary and predominant genes in meeting current production demands, there is an urgent necessity to explore novel genes and proteins (subtypes) to enhance the meat production traits of yellow-feathered broiler chickens. Therefore, in this study, we utilized a high-throughput sequencing strategy to identify circRNAs with translational capacity in myoblasts. We validated that a specific circRNA, termed circSIK2, which is regulated by N6-methyladenosine for translation, has the ability to encode SIK2-176aa and thereby facilitate muscle development and hypertrophy. We were hoping that our existing data and findings could provide more potential coding circular RNAs and functional proteins for poultry muscle development.

## Introduction

In poultry, the hypertrophy of muscle fibers can directly influence the yield of meat products, and this process is regulated by numerous genes, non-coding RNAs, and circular RNAs [1–3]. The proliferation and differentiation of myoblasts before birth play a crucial role in determining both the number and diameter of muscle fibers [4]. CircRNAs were first discovered more than 40 years ago in viroids of higher plants [5], but they attracted little attention until recently, when deep sequencing revealed a large number of circRNAs across multiple species. CircRNAs can originate from exons, introns, untranslated regions, as well as intergenic regions through a back-splicing reaction [6]. Generally, circRNAs are considered a new class of non-coding RNAs (ncRNAs) because they lack polyadenylated (polyA) tails, are expressed at relatively low levels, and play critical roles in gene regulation by acting as the sponges to neutralize miRNA [7,8]. Although the miRNA “decoy” function of circRNAs is well characterized, only a few circRNAs contain perfect miRNA binding sites, suggesting that circRNAs might have additional functions, yet undiscovered, functions.

An intriguing unanswered question is whether circRNAs can be translated into proteins. The possibility arises because most circRNAs originate from gene exons, possess a complete open reading frame (ORF), and are localized in the cytoplasm. Indeed, some RNAs previously classified as “non-coding”, such as long non-coding RNA (lncRNA) [9,10], and microRNAs (miRNAs) [11], have been shown to be translated into functional peptides. To date, four circRNA translation mechanisms have been identified: rolling circle amplification (RCA) translation [12,13], internal ribosomal entry site (IRES) dependent (IRES-dependent) translation [14], IRES-independent translation [15], and N6-methyladenosine (m^6^A)-dependent translation [16]. In 1995, Chen et al. reported that artificially engineered circRNAs containing IRES elements could encode proteins [14]. In 2017, Yang et al. demonstrated that m^6^A, the most abundant RNA base modification, promotes efficient initiation of protein translation from circRNAs in human cells [16]. Circular RNAs contains an infinite open reading frame that can be efficiently translated into proteins in *E. coli,* similar to the rolling circle amplification observed in DNA polymerase reaction [12]. RCA translation of circRNAs not only produces long, repeating peptide sequences but also enhances the production of abundant protein products over time, as the ribosome does not need to repeatedly rebind to the RNA template. Notably, RCA translation of circRNAs occurs without the need for specific elements such as IRES, polyA tail, or cap structure [12,13,15].

Since 1995, many studies have demonstrated that artificial circRNAs can be translated into proteins [14]. Additionally, endogenous protein-coding circRNAs have been reported continuously every year since 2017 [17–22]. However, most of these translations are driven by IRES elements [23]. To date, only two instances of circRNA translation mediated by m^6^A have been reported [16,24]. The m^6^A modification process is catalyzed by a methyltransferase complex composed of METTL3, METTL14, WTAP, and other proteins [25–28]. The fate of methylated RNA is determined by the recruitment of specific reader proteins. The YTH family is a well-known group of m^6^A reader, with different YTH family proteins regulating various process, including RNA export [29], degradation [30], and translation [30,31]. HNRNPA2B1 is another m^6^A reader that has been reported to regulate alternative splicing events [32]. Additionally, HNRNPC and HNRNPG, two other proteins from the HNRNP family, have been reported to regulate the transcript of methylated RNA [33,34].

CircRNA can regulate the growth and development of skeletal muscle in various ways. For example, as non-coding RNAs, they can act as miRNA sponge, thereby releasing downstream lncRNAs or mRNAs [35–37], or they can form circRNA-protein complex [38,39] to regulate myogenesis. Two circRNAs (circZNF609 and circFAM188B) have been reported to have coding potential during myogenesis, and both of their translations are driven by IRES elements [40,41]. Therefore, exploring the translatable circRNAs and their mechanism in poultry skeletal muscle is of great significance for gaining a better understanding of the myogenesis process.

So far there is no general method for isolating and identifying protein-coding circRNA due to the following reasons. First, only a few databases efficiently identify IRES elements or evolutionarily conserved ORFs of circRNA. Second, most exonic circRNAs are originated from mRNA exons; therefore, these ORFs may overlap with their hosting mRNAs, leading to difficulties in distinguishing whether the protein was coded by circRNA or host mRNA. Third, there is a lack of specific antibodies to detect circRNA coding proteins *in vivo*. So, in this study, we were trying to combine circular RNA sequencing, ribosome profiling, and nano-HPLC-MS/MS to explore circRNAs with translation potential in myoblasts, identify the translation mechanism of circSIK2 regulated by m^6^A, and find out the regulatory role of circSIK2 in skeletal muscle development.

## Results

### Overview of circular RNA deep sequencing data

**Fig 1:**
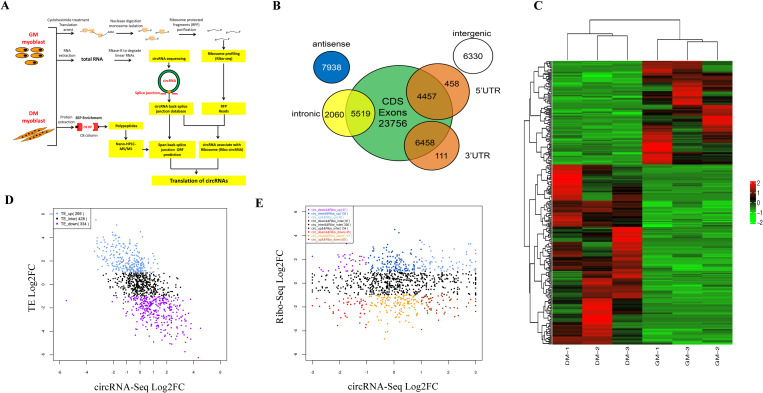
Overview of circRNA-Seq and Ribo-Seq data. (A) A dedicated strategy following high throughput methods (including circRNA-seq, ribosome profiling, and HPLC-MS/MS) was integrated to identify coding circRNA. CircRNA-seq was first performed to identify the circRNA in chicken myoblasts (GM; *n* = 3) and myotubes (DM; *n* = 3). Second, RPFs of ribosome profiling were searched against the circRNA back-splice junction sequences database to identify ribosomal associated circRNA. Third, the MS/MS spectra were searched against a custom protein database from span back-splice junction circRNA ORF prediction. (B) Venn diagram represents the number of circRNA originating from different genomic regions, including exons, intron, exon-intron, intergenic regions, or antisense. (C) Heat map of differentially expressed circRNA between GM and DM group. (D)Translation efficiency (TE) comparison of ribo-circRNA in GM and DM group. FC means fold change. The TE was calculated by $\frac{Ribo-seq\;expression}{CircRNA-seq\;expression}$. (E) Conjoint analysis in transcriptome and ribosome level of ribo-circRNA in GM and DM group.

In this study, we developed an effective strategy to identify protein-coding circRNA by combining circular RNA sequencing, ribosome profiling, and nano-HPLC-MS/MS (Fig 1A). To characterize circRNA transcripts, we performed RNA-seq analyses of ribosomal and linear RNA-depleted total RNA. Total RNA was collected from three replicate samples of chicken myoblasts cultured in growth medium (GM; **n* *= 3) or induced to differentiate (in differentiation medium) into myotubes (DM; *n* = 3). A total of 40,084 circRNAs were identified; 23,434 or 27,450 circRNAs were detected in GM (myoblasts) or DM (myotubes) group, respectively (S1 Table). Unique mapped reads approximately ranging from 74%-80% (S2 Table). These results were consistent with the scope and abundance of identified mammalian circRNAs [42,43]. The length distribution of back-spliced reads varied from 2 bp to 395 bp (S1A Fig). According to their genomic locus, chicken circRNAs were grouped as exon [including 5′ untranslated region (5′ UTR), 3′ UTR, coding sequence (CDS)-5’UTR, and CDS-3’UTR], intron, exon-intron, intergenic regions, or antisense (Fig 1B). Although circRNAs can arise from almost any location in a genome, they mostly originated from coding exons (Fig 1B). The length distribution analyses revealed most identified circRNAs were less than 5,000 nucleotides (nt) in sequence length (S1B Fig). Chromosome distribution analysis demonstrated there were no obvious differences between GM and DM circRNA, but the total number of expressed circRNAs were slightly greater in the DM group (S1C and S1D Fig). As shown in Fig 1C, more than half of the differential expressed circRNAs in the GM and DM groups had different circRNA expression patterns.

Approximately 85% (33,754) of all circRNAs (40,084) were originated from 7,391 chicken genes, especially in mRNA exon region (**n* *= 18,237). Among them, 2,381 parental genes generated only one circRNA, and other genes could yield two or more circular isoforms (S1 Table), similar to the results in pigs [42]. Although most circRNAs were generated from mRNA, the characteristic distinction between circRNA and their host mRNA remains unknown. Full-length distribution analysis revealed most of the mRNA-originated circRNAs were less than 5,000 nt, while their host mRNAs were within 7,500 nt (S2A Fig and S3 Table). Exon numbers of the majority of mRNA-originated circRNAs were less than 20, but host mRNAs were within 30 (S2B Fig and S4 Table). Although host mRNAs were longer (S2A Fig) and have a greater quantity of exons than circRNAs (S2B Fig), surprisingly they have similar CDS length distribution (S2C Fig and S5 Table). The CDS length of host mRNAs were just slightly longer than circRNAs. This finding revealed that circRNAs had a similar protein translation template content compared to host mRNAs, indicating they may have coding potential.

In eukaryotes, translation initiation of mRNA by the 40S ribosomal subunit occurs in the vicinity of the 5′ cap structure of the mRNA [43]. Because circRNAs do not contain a 5’ cap like mRNAs, their translation should rely on the presence of a translation regulatory element. Picornaviruses can initiate translation via an internal ribosome entry site (IRES), an RNA structure that directly recruits the 40S ribosomal subunits in a cap-independent fashion [44]. Previous studies revealed internal ribosomal entrance elements in UTRs of circRNA allow cap-independent translation [17,18]. Our study discovered that circRNAs have similar UTR length distribution with the host mRNAs (S2D Fig and S6 Table). The UTR of circRNA may have contained IRES, which enabled the initiation of cap-independent translation of circRNA ORF. In general, mRNA-originated circRNAs and their host mRNAs have similar properties in the length of CDS and UTR, but they were different in their full-length and exon numbers. This finding revealed chicken circRNA had similar length distribution of CDS and UTR with their host mRNA.

### Discovering ribosomes-associated circRNA

To study the specific coding possibility of chicken circRNAs, we identified those with open reading frames spanning a back splice junction. We found a total of 15,332 circRNAs ORF (cORF; S7 Table). There were 3,927 of them without a stop codon and the number of nucleotides composing the RNA is a multiple of three (S8 Table; referred hereafter as MOEBIUS_ORF circRNA). After annotating chicken cORF, we searched for evidence of their translation. We utilized ribosome profiling (Ribo-seq) to monitor the translation event in chicken myoblasts and myotubes. Ribosomal protected fragments (RPF) were searched against a circRNA back-splice junction sequence database to identify ribo-circRNAs. A total of 4,069 ribo-circRNAs with at least one specific RFP read were identified (S9 Table). We also used the IRESfinder tool to predict which circRNAs contained IRES, and 3,471 of the 4,069 ribo-circRNAs had the IRES element (S10 Table). It is noteworthy that this result was obtained again using rigorous screening criteria for only the RPF containing across back-splice junctions that were considered as ribo-circRNAs reads. In addition, 860 MOEBIUS_ORF circRNAs were found with at least one specific RFP (S11 Table). This result suggests the translation of MOEBIUS_ORF circRNAs in chicken myoblasts is more probable than it was previously thought to be. We combined the ribo-circRNAs with the circRNAs found in circular RNA-seq and calculated their translation efficiency (TE), there were 269 ribo-circRNAs had higher TE, and 334 ribo-circRNAs lower in GM than the DM group (Fig 1D). We also compared the transcriptome level and ribosome level of ribo-circRNAs, only 69 ribo-circRNAs in GM had both higher transcriptome and ribosome levels than the DM group, and 45 ribo-circRNAs had both lower transcriptome and ribosome levels than DM group (Fig 1E).

### Analysis of endogenous peptide translated from circRNAs junction

**Fig 2:**
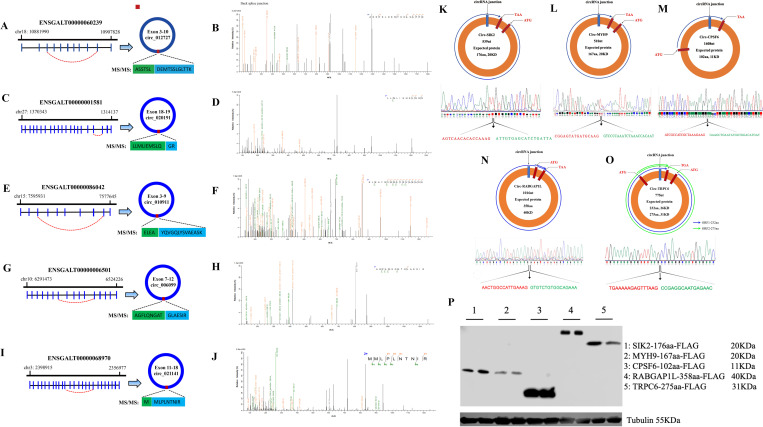
MS/MS identified the circRNA spanning back-splice junction-coded endogenous peptides. (**A, B**) The high energy collisional dissociation (HCD) MS/MS spectrum and origination of circ_012727 peptide **ASSTSLDEMTSSLGLTTK**. (**C, D**) The high energy collisional dissociation (HCD) MS/MS spectrum and origination of circ_020191 peptide **LLMLIEMSLQGR**. (**E, F**) The high energy collisional dissociation (HCD) MS/MS spectrum and origination of circ_010911 peptide **ELEAYQVGQLYSVAEASK**. (**G, H**) The high energy collisional dissociation (HCD) MS/MS spectrum and origination of circ_006099 peptide **AGFLQNGATGLAESIR**. (**I, J**) The high energy collisional dissociation (HCD) MS/MS spectrum and origination of circ_021141 peptide **MMLPLNTNIR**. (**K-O**) The putative open reading frame (ORF) (upper panel) and the Sanger sequencing validation (lower panel) of circSIK2, circMYH9, circCPSF6, circRABGAP1L, and circTRPC6. (**P**) Flag tag antibody was used to detect the putative ORF expression of circSIK2, circMYH9, circCPSF6, circRABGAP1L, and circTRPC6 in DF-1 cell lines.

To directly identify endogenous peptides translated from circRNAs junctions, we created a customized database (S12 Table) containing peptides encoded by RNA sequences spanning back-splice junctions of all circRNAs and combined them with the UNIPROT Chicken database (*Gallus gallus*). Peptides obtained from nano-HPLC-MS/MS across back-splice junction were then filtered against the UNIPROT chicken database using a string-searching algorithm to ensure these peptides were not from any known chicken protein. We identified 5 peptides encoded by the back splice junctions of 5 circRNAs that do not match any known proteins from the UNIPROT chicken database (Fig 2). The collision-induced dissociation MS/MS spectrum from 5 circRNAs back splice junctions was shown (Fig 2A–2J). Surprisingly, circ_012727 (Fig 2A and 2B) did not have any stop codons, and thus, theoretically never terminated. We also identified another 5 circRNAs (Fig 2K–2O) by sanger sequencing (primer was shown in S13 Table) and their encoding peptides by cloning their ORF sequences into pcDNA3.1 vector with a Flag tag. Those vectors were transfected into DF-1 cell lines, which expressed the peptides encoded by circRNAs (Fig 2P). We analyzed the conservativeness of those 10 encoding circRNAs and found out most of them were highly conservative among birds (S3 Fig). These peptides merely represented a small fraction of the circRNA-encoded proteome, because only the peptide sequence spanning the back splice junction can be unambiguously identified as circRNA-encoded products and a lot of verification work needs to be done to validate the coding ability of ribo-circRNAs.

### CircSIK2 differential expressed in myogenesis and was translatable

**Fig 3:**
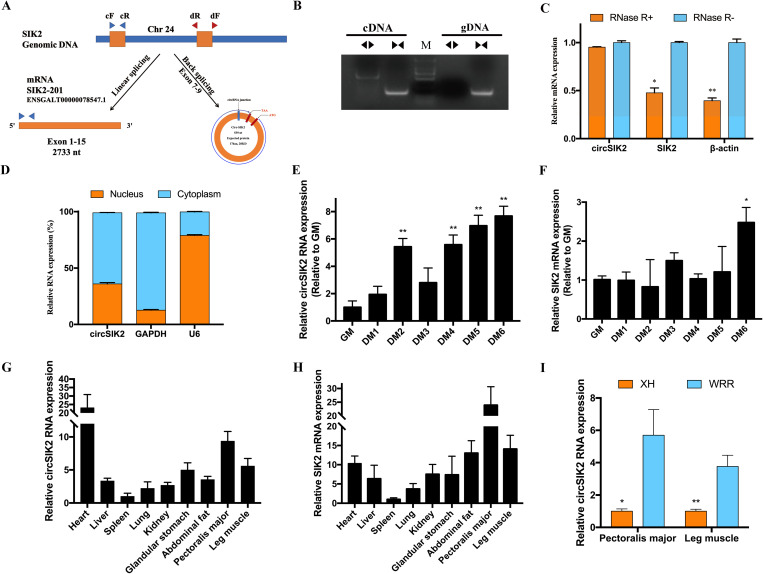
Identification of CircSIK2. (**A**) The schema of circSIK2 is derived from the exons 7-9 of the SIK2 gene. (**B**) Divergent primer amplified circSIK2 in cDNA but not gDNA, convergent primer was used as a positive control. (**C**) qRT-PCR results of circSIK2, SIK2, and β-actin mRNA after RNase R digestion. (**D**) CircSIK2 is localized mainly in the cytoplasm of myoblast cells. GAPDH and U6 serve as cytoplasmic and nuclear localization controls respectively. (**E-F**) The expression pattern of circSIK2 (**E**) and SIK2 (**F**) in different stages of myoblast differentiation. GM (growth media; *n* = 3) stands for myoblasts in the proliferative phase. DM (differentiation media; *n* = 3), DM1-DM6 means differentiation from day 1 to day 6. (**G-H**) Tissue expression profiles of circSIK2 (**G**) and SIK2 (**H**) in 7 weeks old WRR chicken (*n* = 4). (**I**) Relative circSIK2 expression in pectoralis major and leg muscle between 7 weeks old WRR and XH chicken (**n* *= 6).

CircSIK2 is one of the circRNAs that has been validated as having coding potential (Fig 2K and 2P), which originated from the exons 7, 8, and 9 of its parent gene SIK2 with a total length of 539 nt (Fig 3A). The PCR result amplified by convergent primer (cF/R) and divergent primer (dF/R) shows that divergent primer gets a single distinct band only in the cDNA sample along with the Sanger sequencing (Figs 2K and 3B) matched to the back spliced sequence indicating the real existence of circSIK2, the RNase R treatment also confirmed the stability of circSIK2 compared with the linear SIK2 mRNA (Fig 3C). The cellular localization results showed circSIK2 exists in both the nucleus and cytoplasm, but more in the cytoplasm (Fig 3D). Although circSIK2 was not differentially expressed during GM and DM3, which is consistent with our previous circRNAs sequencing data, it was significantly upregulated in other differentiation periods like DM2, DM4, DM5, and DM6 (Fig 3E) while the parent gene SIK2 was only showed significantly upregulated at DM6 (Fig 3F). White Recessive Rock (WRR) and Xinghua chicken (XH) has been used as good models to study muscle hypertrophy due to their significant body weight difference at 7 weeks of age, which could be up to 5 times [45,46]. Tissue expression profiles of circSIK2 and SIK2 in 7-week-old WRR chicken showed that both circSIK2 and SIK2 showed relatively high expression in heart, pectoralis major, and leg muscle (Fig 3G–2H), and compared with XH chicken, circSIK2 was also significantly upregulated in muscle tissues of WRR chicken (Fig 3I).

**Fig 4:**
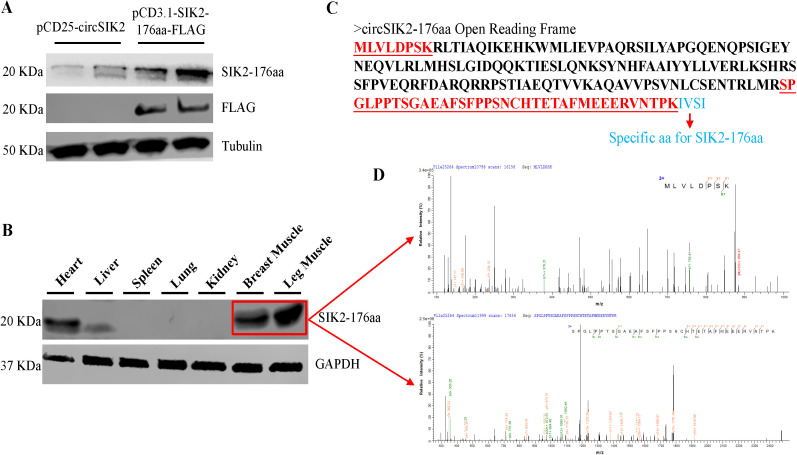
The translatable of circSIK2. (**A**) SIK2-176aa specific antibody and FLAG tag antibody were used to detect the SIK2-176aa expression of pCD25-circSIK2 and pCD3.1-SIK2-176aa-FLAG vector in myoblast cells. (**B**) The expression of SIK2-176aa was detected in 7 weeks old WRR chicken heart, liver, breast, and leg muscle tissues. (**C**) The predicted SIK2-176aa peptide sequence. (**D**) Two SIK2-176aa peptides were identified and showed (Upper: **MLVLDPSK**. Lower: **SPGLPPTSGAEAFSFPPSNCHTETAFMEEERVNTPK**.).

Previous results indicated circSIK2 potentially encoded a 176aa (we named as SIK2-176aa) peptide (Fig 2K and 2P), both the pCD25-circSIK2 and pCD3.1-SIK2-176aa-FLAG vector could successfully overexpress the SIK2-176aa peptide in myoblast cells (Fig 4A). Western Blot could not detect the SIK2-176aa, probably due to the low protein abundance in the myoblast cells. However, we successfully found this protein in the heart, liver, breast, and leg muscles of a 7-weeks old WRR chicken (Fig 4B). We further confirmed the existence of SIK2-176aa by sending the isolated 20 kDa coomassie blue SDS-PAGE gel from breast and leg muscle tissue for LC-MS/MS and the identified amino acid sequences covered 61% and 92% of SIK2-176aa, the first and last match of two fragments were shown in Fig 4C and 4D.

### CircSIK2 promoted myoblast cell cycle progression and myotube formation

**Fig 5:**
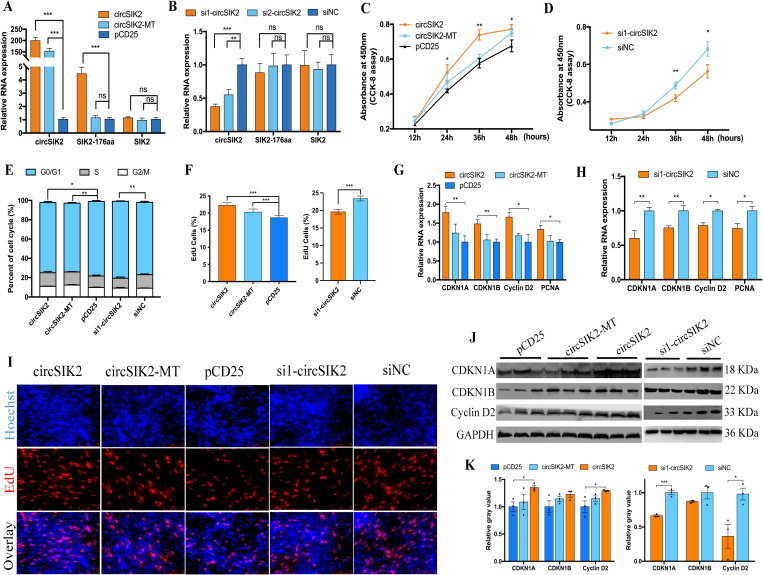
CircSIK2 promotes the cell cycle progression of myoblasts. (**A, B**) The transfection efficiency of pCD25-circSIK2, pCD25-circSIK2-MT(**A**), and siRNAs (**B**) for circSIK2 (*n* = 4). (**C, D**) CCK-8 assay was performed to assess the effect of circSIK2 overexpression and knockdown on myoblast proliferation (*n* = 8). (**E**) The statistical results of cell cycle analysis after overexpression and inhibition of circSIK2 in myoblast (*n* = 4). (**F, I**) The proliferation rate (**F**) and EdU staining (**I**) of myoblast cells transfected with pCD25-circSIK2, pCD25-circSIK2-MT, and si1-circSIK2 (*n* = 4). (**G, H**) mRNA level of *CDKN1A*, *CDKN1B*, *Cyclin D2*, and *PCNA* after overexpression and knockdown of circSIK2 (*n* = 4). (**J, K**) The protein expression of CDKN1A, CDKN1B, and Cyclin D2 (**J**) and their relative grey value (**K**) was determined by western blot in myoblast cells (*n* = 3), western blot was from an aliquot of the same sample but were run and blotted from different gels (*P < 0.05; **P < 0.01, ***P < 0.001).

To investigate the role of circSIK2 in chicken primary myoblast activity, we constructed the overexpression plasmid and small interfering RNA (siRNAs) for circSIK2, the circSIK2-MT plasmid (mutant the initiator codon ATG of circSIK2 into ACG) was used to rule out the translation of SIK2-176aa peptide. All those plasmids and siRNA could successfully improve or knockdown the expression of circSIK2, but not the liner SIK2 gene (Fig 5A and 5B). Flow cytometry showed circSIK2 overexpression arrested G0/G1 progression and increased the number of cells in the S phase (Fig 5E). As expected, the CCK-8 assay (Fig 5C and 5D) and EdU assay (Fig 5F and 5I) showed enhanced cell growth and cell proliferation after circSIK2 overexpression and an opposite trend when circSIK2 was knocked-down. Also, mRNA and protein levels of cell cycle related genes like CDKN1A, CDKN1B, and Cyclin D2 were increased after myoblasts were transfected with circSIK2 (Fig 5G, 5J and 5K) and decreased with circSIK2 siRNA (Fig 5H, 5J and 5K).

**Fig 6:**
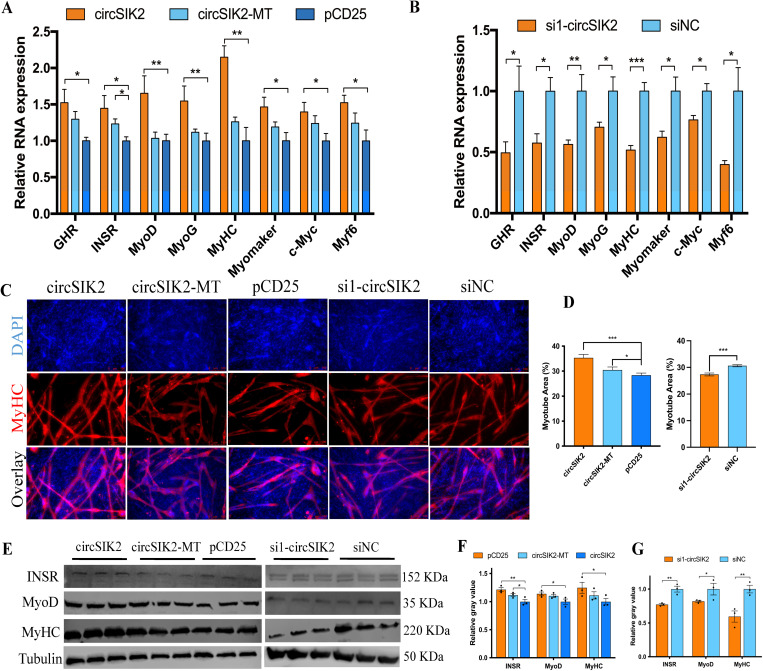
CircSIK2 facilitates myotube formation. (**A, B**) The relative RNA expression of myoblast cell differentiation associated genes, including *GHR*, *INSR*, *MyoD*, *MyoG*, *MyHC*, *Myomaker*, *c-Myc*, and *Myf6* after circSIK2 overexpression and inhibition. (**C, D**) MyHC staining (**C**) and myotube area (**D**) of cells after transfection with pCD25-circSIK2, pCD25-circSIK2-MT, and si1-circSIK2. Myoblasts were expanded in growth medium until the designated confluence (when the siNC transfected cells reached approximately 90% confluence) was reached. Differentiation was then induced by replacing the growth medium with differentiation medium for 3 days. The resulting myotubes were fixed and analyzed. (**E-G**) The protein level (**E**) and relative grey value (**F-G**) of INSR, MyoD, and MyHC after overexpression and knockdown of circSIK2, western blot was from an aliquot of the same sample but were run and blotted from different gels (*P < 0.05; **P < 0.01, ***P < 0.001).

Besides myoblast cell proliferation, we also want to know the function of circSIK2 in myoblast differentiation. Therefore, we performed the MyHC immunofluorescence staining and showed the number and area of myotubes were greatly increased with circSIK2, while knockdown of circSIK2 suppressed the formation of myotubes (Fig 6C and 6D). qRT-PCR and western blot results showed circSIK2 stimulated the expression of *GHR*, INSR, and myogenic regulatory factor family members like MyoD, *MyoG*, MyHC, *Myomaker*, *c-Myc*, and *Myf6* (Fig 6A, 6B and 6E–6G). Although the trend was consistent with circSIK2, some genes were not affected by circSIK2-MT plasmid (Figs 5G and 6A), and when the SIK2-176aa peptide was not expressed, the promotion of myoblasts proliferation and differentiation declined (Fig 5 and 6). A liner exon 7–9 transcript (encode circSIK2) was constructed to rule out the liner forms of SIK2 masquerade. This control experiment specifically rules out potential interference from liner SIK2 transcripts (S4 Fig). Our results demonstrated that while high-level overexpression of these linear fragments can induce minimal circSIK2 production via reverse splicing, this effect: (a) does not significantly alter the expression of proliferation or differentiation related genes, and (b) fails to produce circSIK2-derived proteins. These results suggest that circSIK2 may play its role in facilitating myogenesis by the SIK2-176aa peptide.

### CircSIK2 regulated myoblast proliferation and differentiation, and muscle fiber hypertrophy through SIK2-176aa

**Fig 7:**
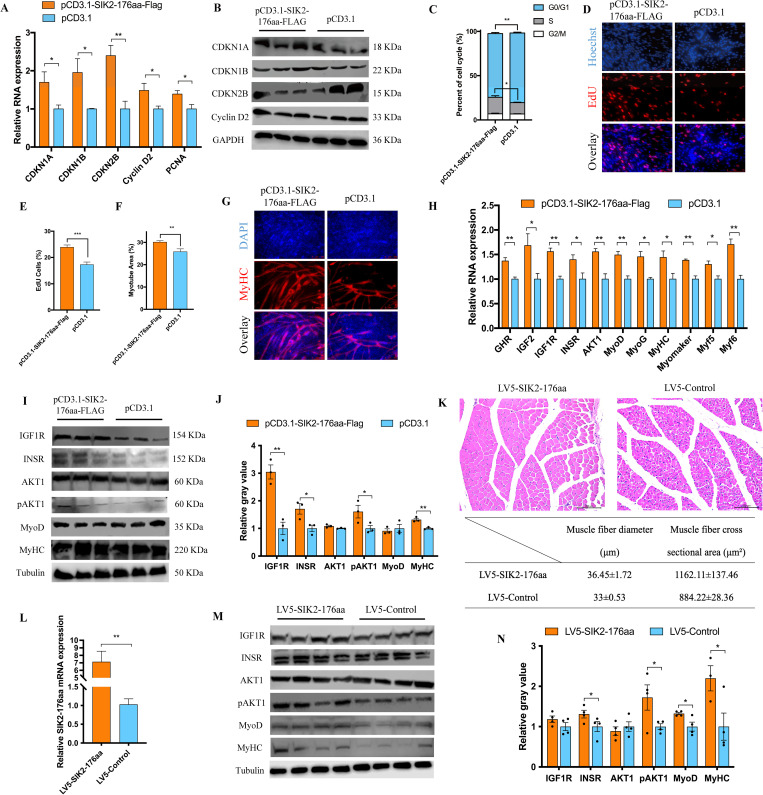
SIK2-176aa regulates myoblasts proliferation and differentiation and muscle fiber hypertrophy. (**A**) SIK2-176aa promotes the mRNA level of *CDKN1A*, *CDKN1B*, *CDKN2B*, *Cyclin D2*, and *PCNA*. (**B**) The protein expression of CDKN1A, CDKN1B, CDKN2B, and Cyclin D2 was determined by western blot in myoblast cells. (**C**) The statistical results of cell cycle analysis after overexpression of SIK2-176aa. (**D, R**) The EdU staining (**D**) and proliferation rate (**E**) of myoblast cells transfected with pCD3.1-SIK2-176aa-FLAG. (**F, G**) Myotube area (**F**) and MyHC staining (**G**) after transfected with pCD3.1-SIK2-176aa-FLAG. (**H**) SIK2-176aa promotes the mRNA level of GHR-IGF-AKT pathway genes including *GHR*, *IGF2*, *IGF1R*, *INSR*, *AKT1*, and the myogenic regulatory factor family member including *MyoD*, *MyoG*, *MyHC*, *Myomaker*, *Myf5*, and *Myf6*. (**K**) H-E staining of breast muscle fiber cross section infected with LV5-SIK2-176aa or LV5-Control (upper panel) and the statistical data of muscle fiber diameter and cross-sectional area (lower panel). Lentivirus was injected three times into the pectoral muscles of 1-day-old chicks (n = 10) at days 1, 4, and 7 at a dosage of 1 × 10^8^ IU/mL. The pectoral muscles were obtained 14 days after the first injection. (**L**) q-RT PCR detection of the injection efficiency of LV5-SIK2-176aa. (**I-J, M-N**) SIK2-176aa promotes the protein expression of IGF1R, INSR, p-AKT1, MyoD, and MyHC *in vivo* (**I-J**) and *in vitro* (**M-N**), western blot was from an aliquot of the same sample but were run and blotted from different gels.

To verify that circSIK2 played its role by encoding SIK2-176aa, we further validated the role of SIK2-176aa individually. Due to the low abundance of proteins expressed by the pCD25-circSIK2 vector and the instability of SIK2-176aa specific antibody, we chose to use the pCD3.1-SIK2-176aa-FLAG vector to overexpress the SIK2-176aa peptide with FLAG tag (Fig 4A). As expected, SIK2-176aa showed the same trend with circSIK2 and promoted cell cycle progression of myoblasts through CDKN1A, CDKN1B, CDKN2B, Cyclin D2, and PCNA (Fig 7B–7E). Myosin heavy chain staining also showed the SIK2-176aa could accelerate the myotube formation (Fig 7F–7G). The qRT-PCR and western blot results showed SIK2-176aa could stimulate myoblast differentiation through the GHR-IGF-AKT pathway and myogenic regulatory factor family members like MyoD, *MyoG*, MyHC, *Myomaker*, *Myf5*, and *Myf6* (Fig 7H–7J). The *in vivo* lentiviral intramuscular injection of SIK2-176aa could effectively increase the expression of SIK2-176aa mRNA level (Fig 7L) and promote muscle fiber hypertrophy by increasing muscle fiber diameter and cross-sectional area (Fig 7K) through GHR-IGF-AKT pathway and myogenic regulatory factor family members like MyoD and MyHC (Fig 7M and 7N).

### N6-methyladenosine mediated the translation of circSIK2

**Fig 8:**
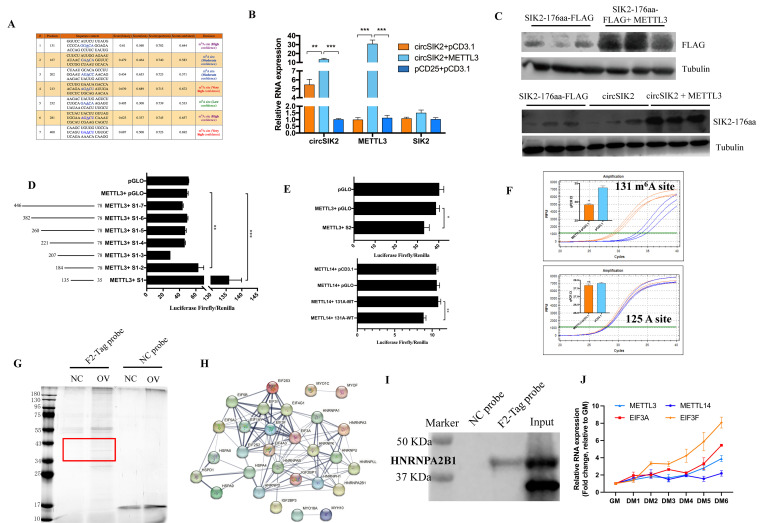
m^6^A mediates the translation of circSIK2. (**A)** Details of seven potential methylation modification sites on circSIK2. **(B**) The relative expression of circSIK2, METTL3, and SIK2 mRNA in myoblast cells after transfected with pCD25-circSIK2 + pCD3.1, pCD25-circSIK2 + pCD3.1-METTL3, and pCD25 + pCD3.1. (**C)** Upper: the expression of SIK2-176aa-flag after transfected with pCD3.1-SIK2-176aa-flag and pCD3.1-SIK2-176aa-flag + pCD3.1-METTL3. Lower: the expression of SIK2-176aa after transfected with pCD3.1-SIK2-176aa-flag, pCD25-circSIK2, and pCD25-circSIK2 + pCD3.1-METTL3. **(D**) Dual-luciferase reporter assay using the different regions of the circSIK2 after co-transfected with METTL3 in DF-1 cells. (**E)** Upper: Dual-luciferase reporter assay using the 151–200 fragment after co-transfected with METTL3 in DF-1 cells. Under: Dual-luciferase reporter assay after 131A-WT and 131A-MT fragment was co-transfected with METTL14 in DF-1 cells. **(F**) Amplification curve and qPCR CT value in circSIK2 131 m^6^A site and 125 A site after METTL3 overexpression in myoblast cells. (**G, H)** circSIK2 pulldown image (**G**) (OV = pCD25-circSIK2, NC = pCD25) and binding proteins’ string network (**H**) screened by mass spectrometry in myoblast cells. (**I**) circSIK2 pulldown results confirmed by western blot with HNRNPA2B1 antibody in myoblast cells. (J) The expression pattern of METTL3, METTL14, EIF3A, and EIF3F in different stages of myoblast differentiation. GM (growth media; *n* = 3) stands for myoblasts in the proliferative phase. DM (differentiation media; *n* = 3), DM1-DM6 means differentiation from day 1 to day 6.

Previous IRESfinder results (S10 Table) showed there were 5 fragments in circSIK2 that may contain IRES elements, but we didn’t find an IRES-induced Luc/Rluc activity in our luciferase assay, thus we speculate that the translation of circSIK2 was regulated by other mechanisms like m^6^A. Through SRAMP (sequence-based RNA adenosine methylation site predictor, http://www.cuilab.cn/sramp) prediction server we obtained 7 potential m^6^A modification sites (Fig 8A), and METTL3 could significantly promote the expression of circSIK2 and SIK2-176aa peptide without affecting the expression of SIK2 in myoblast cells (Fig 8B and 8C). The specificity of SIK2-176aa antibody was also detected in DF-1 cells by transfecting METTL3 + circSIK2, circSIK2, circSIK2-MT (mutant the initiator codon ATG of circSIK2 into ACG), SIK2-176aa-FLAG, results showed that the specificity of SIK2-176aa antibody in DF-1 cells is better than in chicken primary myoblast cells and chicken tissues, and METTL3 could promote the expression of SIK2-176aa in DF-1 cells (S5 Fig). This result suggests that METTL3 could modify the m^6^A sites on circSIK2, promote the transcription of circSIK2, and then drive the translation of circSIK2. To locate the specific modified m^6^A site recognized by METTL3, we conducted a Dual-Luciferase reporter assay in DF-1 cell lines. Seven fragments containing 1–7 m^6^A sites were cloned into pmirGLO vector and co-transfected with METTL3, the Luc/Rluc activity of and pGLO-S1-2 were significantly higher than other groups (Fig 8D), suggesting the modified m^6^A sites were at 131 or 167. So, we constructed the pGLO-S2 (151–200, containing 167 m^6^A site) vector and co-transfected with METTL3, the result showed that the transcriptional activity of the fragment was inhibited in the presence of METTL3 (Fig 8E). Therefore, we determined that the methylation site of circSIK2 recognized by METTL3 was located at the 131 bases of circSIK2. METTL14 was also co-transfected with 131A-WT (100–150, containing 131 m^6^A site) and 131A-MT (131 m^6^A mutation), the dual-luciferase reporter assay results (Fig 8E) showed that METTL14 could also participate in the methylation modification of circSIK2. This result proves that METTL3 could form classical methyltransferase with METTL14, collaboratively participate in the m^6^A modification of circSIK2. We also performed further validation by using the SELECT method to confirm the site-specific m^6^A modification level of circSIK2 and METTL3 in myoblast cells. The N-site at 6 bases upstream of the predicted site was set as a negative control. Only sites 131 showed a significant m^6^A modification level after METTL3 overexpression (Fig 8F), which is consistent with the luciferase assay results (Fig 8D and 8E). Moreover, we performed a circSIK2 RNA pulldown assay in myoblast cells to dig the downstream ‘Reader’ after circSIK2 had been methylated (Fig 8G), and the mass spectrometry results showed that these proteins potentially bind to circSIK2 were enriched in translation initiation, translation, alternative mRNA splicing, and RNA degradation (Fig 8H and S14 Table), and some proteins were related to myogenesis and heat stress. The western blot results also proved that HNRNPA2B1, an m^6^A Reader, could directly interact with circSIK2 (Fig 8I). Based on the expression pattens of METTL3, METTL14, EIF3A, EIF3F, and circSIK2 (Figs 8J and 3E), we believe that the m^6^A modification may have started from early differentiation stage DM2, but the translation mediated by METTL3 was more active from the late differentiation stage DM4–6.

**Fig 9:**
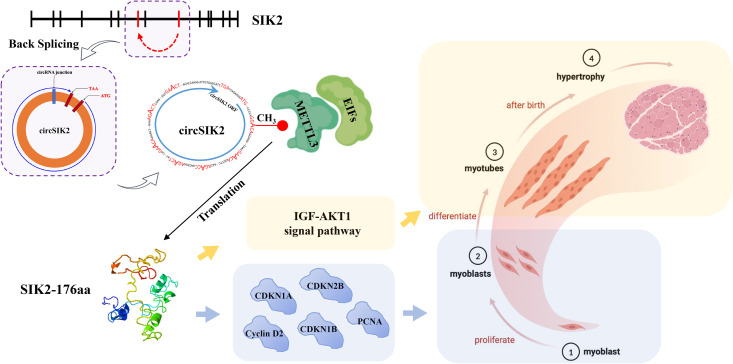
Model of circSIK2 promoting myoblasts proliferation, differentiation, and muscle fiber hypertrophy by translating SIK2-176aa.

In summary, METTL3 could modify the methylation of circSIK2 on site 131 thus promoting its transcription and translation (Fig 9).

## Discussion

It was previously thought that circRNAs can be translated only if they contained an internal ribosome entry site [18] or m^6^A modifications [16]. However, in this study, the predicted IRES elements in circSIK2 didn’t show any differential Luc/Rluc activity. Therefore, we speculated that the translation of circSIK2 may occur due to the m^6^A modification and found seven potential m^6^A sites in circSIK2 (Fig 8A). Through further verification, we found that METTL3 (methyltransferase like 3), an RNA methyltransferase, could methylate the A at the 131st base of circSIK2, and promote the expression of both circSIK2 and SIK2-176aa. Although METTL3 is famously known as a member of the methyltransferase complex and interacts with METTL14 and WTAP, our results clearly show that the function of METTL3 here is dual, forming classical methyltransferase with METTL14, and direct translational activator. Research has shown that different cellular localization of METTL3 may lead to different functions [47]. When METTL3 is in the cytoplasm, it could recruit translation initiation factors like eIF3h to directly promote translation independently of the catalytic activity of downstream reader proteins [47–49]. Interestingly, the circSIK2 pulldown didn’t directly enrich the METTL3 protein but pulled down some translation initiation proteins (Fig 8H and S14 Table). Considering the low protein abundance of SIK2-176aa in the myoblast cells and the expression pattens of METTL3, METTL14, EIF3A, EIF3F, and circSIK2, we believed that the m^6^A modification of circSIK2 may have started from early differentiation stage DM2, and accumulation of SIK2-176aa might be initiated when the methylation level is relatively high and METTL3 is highly expressed at late differentiation stage DM4–6. During myoblast differentiation, the high expression of circSIK2 coincided with the high methylation of myoblasts [50], indicating that circSIK2 has high translation potential during this process. The fact that SIK2-176aa was found in hypertrophic broilers, but not in leaner broilers (Xinghua chicken), was consistent with the results showing that SIK2-176aa promoted myoblast proliferation, differentiation, and muscle hypertrophy. It’s limited to attribute hypertrophy solely to SIK2-176aa in a localized model, the intramuscular delivery method does suggest localized action, but we acknowledge potential confounding factors (e.g., inflammation-induced hypertrophy or minor peptide diffusion). Future studies may use systemic delivery (e.g., IV or IP injection of LV-expressed SIK2-176aa) or new time-course (3, 7, 14, 21 days post-injection) to clarify this.

CircRNAs originating from exonic sequences are abundant in human cells [8,51]. Previously, knowledge of exonic circRNAs was limited to a handful of genes, and circRNAs were considered transcriptional errors or byproducts [52,53]. However, RNA-seq has revealed that circRNAs are more conserved and prevalent [51] in eukaryotes than previously thought. Some exonic circRNAs (eciRNAs) can function as miRNA sponges, competing with mRNA for miRNA binding, and thereby upregulating the expression of miRNA target genes. A representative example is the cerebellar degeneration-related 1 antisense transcript (CDR1as), which contains over 60 conserved miR-7 target sites [7]. Research has reported that circRNAs can simultaneously regulate the proliferation and differentiation of myoblasts by sponging miRNAs and releasing different miRNA target genes, such as circSVIL and circSNX29 [54,55]. Typically, the regulation of myoblast proliferation relies on cell cycle-related proteins, as observed in our findings with circSIK2 and SIK2-176aa. Interestingly, SIK2-176aa appears to influence more cyclin-related proteins than SIK2-176aa alone (Figs 5G, 5H, 7A and 7B). Myoblast differentiation can be affected by various genes, including myogenic regulatory factors such as the MyoD family members (MyoD, Myf5, MyoG, MRF4), Myomaker, c-Myc [1,2,56,57]. Our results suggest that circSIK2 primarily exerts its regulatory role in myogenesis by translating SIK2-176aa, which, in turn, regulates the expression of genes in GHR-IGF-AKT pathway, including *GHR*, *IGF1R*, *IGF2*, *INSR*, and *AKT1*. This regulation contributes to the differentiation and hypertrophy of myoblasts. Although the overall trend was consistent, the function of circSIK2 was not identical to that of SIK2-176aa, possibly due to the simultaneous regulation of circSIK2 by certain miRNAs.

CircRNAs represent a novel class of alternative protein sources with potential to modulate muscle traits in poultry and livestock. Functionally characterized circRNA-derived peptides—including circZNF609 and circFAM188B-133aa (myogenesis regulation), circNEB-907aa (ubiquitination-dependent SKP1 interaction and TPM1-mediated myoblast fusion during proliferation/differentiation in vitro and regeneration in vivo [58]), and circANKRD17–571aa (lipid droplet formation and maturation [59])—demonstrate conserved roles in myogenesis and adipogenesis. Despite these advances, the mechanistic basis of circRNA translation remains poorly understood in livestock species. Notably, mechanistically, only our newly identified circSIK2 (chicken) were reported to utilize m^6^A modification-driven translation to generate functional peptides governing muscle development in livestock. Unlike circZNF609 and circFAM188B [40,41], we did not observe any IRES-induced Luc/Rluc activity in circSIK2. In comparison to the m^6^A-driven translation of circMAP3K4 [24], we accurately identified the methylation modification site and determined that the adenosine at position 131 of circSIK2 can be methylated by METTL3, thereby promoting the expression of both circSIK2 and SIK2-176aa. Our findings represent the first example of m^6^A-mediated modification in circRNA translation during poultry myogenesis.

Previous studies have reported the efficient translation of synthetic repeat FLAG-coding sequences, which lack stop codons, via RCA (rolling-circle amplification) in E. coli [12] and living human cells [15]. However, to date, only one instance of endogenous protein translation driven by this mechanism has been discovered [13]. Generally, the majority of eukaryotic mRNA possess a 5’ cap structure and a 3’ polyA tail [60]. Eukaryotic translation initiation is normally cap-dependent because recognition of the cap is required for the assembly of the initiation complex [61]. In the RCA translation system, circRNA contain no stop codon, and the number of nucleotides composing the RNA is a multiple of three [14,62]. Thus, theoretically, the elongation process can continue indefinitely once translation initiation occurs. The RCA mechanism not only produces a long, repeating peptide sequences but also enhances productivity over a given period of time because the ribosome does not need to rebind to the RNA template multiple times, which is typically the rate-limiting step in the reaction cycle [63]. In this study, Circ_012727, which originates from TMEM94–201 exon 3–10, is inferred to be a molecule with an infinite open reading frame because it has no stop codon and the number of nucleotides composing the RNA is a multiple of three (1,041 nt). RNA-seq only detected a mean density of 1.3 reads in the DM of circ_012727, suggesting it has a very low RNA expression level; however, 18 unique amino acids spanning the back-splice junction of circ_012727 were detected by mass spectrometry. These results indicated that even extremely lowly expressed circRNAs, such as circ_012727, can be efficiently translated in chicken myoblast cells to produce an abundant protein product by via the rolling-circle amplification mechanism. Our previous ribosome profiling results identified a large group of MOEBIUS_ORF circRNAs (S10 Table), as the proteins encoded by the MOEBIUS_ORF circRNAs were extremely large and unpredictable. So far, there is no way to validate the existence of such proteins unless specific antibodies are developed, but we hypothesize that RCA-driven translation exists in chicken myoblasts.

We acknowledge that our detection likely represents only a fraction of existing circRNA-derived peptides due to MS sensitivity limits, particularly for lowly-abundance circRNA. Native peptides exhibit low stability against proteolysis [64], resulting in transient in vivo activity. Even minimal transient translation from circRNA may compete with linear mRNA translation for ribosomal occupancy or translation factors. Consequently, translationally competent circRNAs at low expression levels face additional detection challenges. The peptides we identified may therefore represent short functional peptides (like uORF products) capable of influencing signaling pathways.

## Materials and methods

### Ethics statement

All animal experiments in this study were performed and carried out according to the protocols approved by the Animal Care and Use Committee of South China Agricultural University, China (approval ID: SCAU#2021f074). All efforts were made to minimize the suffering of animal.

### Animal and cell culture

Chicken embryonic fibroblasts (cell line DF-1) were cultured in Dulbecco’s modified Eagle’s medium (Gibco, Grand Island, NY, USA) supplemented with 10% fetal bovine serum (Gibco) and 0.5% penicillin/streptomycin (Gibco) in a humidified atmosphere with 5% (v/v) CO_2_ at 37^o^C.

Chicken primary myoblasts were isolated from E11 chicken leg muscles as previously described [65]. Chicken primary myoblasts were cultured with a growth medium consisting of RPMI-1640 medium (Gibco), 20% fetal bovine serum, and 0.5% penicillin/streptomycin. Differentiation was induced by switching to serum-restricted medium (RPMI-1640 medium, 2% horse serum, and 0.5% penicillin/streptomycin) specifically when the siNC transfected cells reached approximately 90% confluence.

### Circular RNA library construction and deep sequencing

Chicken primary myoblasts were collected at two time points: myoblasts achieving 50% cell confluence (designated GM group) and myoblasts differentiated for 3 days (designated DM group). Each interval included three biological replicates (**n* *= 3). The total RNA of six myoblast samples was successfully extracted using Trizol reagent (Invitrogen, USA), treated with RNase-R to degrade the linear RNA, and purified using RNeasy MinElute Cleanup Kit (Qiagen, GER). Strand-specific libraries were constructed using VAHTS Total RNA-seq (H/M/R) Library Prep Kit (Vazyme, China) according to the manufacturer’s instructions. In brief, ribosomal RNA was removed from total RNA to retain circRNA. Next, the enriched circRNA was fragmented into short fragments using a fragmentation buffer and reverse transcribed into cDNA with random primers. Second-strand cDNA was synthesized by PCR. The cDNA fragments were purified with VAHTSTM DNA Clean Beads, end-repaired, polyA added, and ligated to Illumina sequencing adapters. Second-strand cDNA was digested with uracil-N-glycosylase (UNG) (Cwbio, China), products were purified using VAHTSTM DNA Clean Beads, and libraries were sequenced using Illumina HiSeqTM 2500.

### Annotation of chicken circRNAs

To collect raw sequencing data of each sample, adapter reads, and low-quality reads were removed using Trim Galore (http://www.bioinformatics.babraham.ac. uk/projects/trim_galore/). To remove rRNA reads, the filtered data were mapped to the rRNA database using the short reads alignment tool Bowtie2 [66]. The rRNA removed reads of each sample were mapped to the chicken genome (Gallus_gallus-5.0) using TopHat software [67], respectively. The genome-mapped reads were discarded, and the unmapped reads were then collected for circRNA identification. To find unique anchor positions within the splice site, 20-mers from both ends of the unmapped reads were extracted and aligned to the chicken reference genome (Gallus_gallus-5.0). To distinguish circRNAs, anchor reads that aligned in the inverted direction (head-to-tail) showed circRNA splicing and were then subjugated to locate_circ [7]. Using GU/AG splice locations, the anchor alignments were prolonged such that the full read aligns, and the breakpoints were flanked. A candidate circRNA was identified if it was supported with at least two unique back-spliced reads. The edgeR package (http://www.rproject.org/) was used to identify differentially expressed circRNAs between GM and DM groups. We identified circRNAs with a fold change ≥ 2 and differences between GM and DM groups were considered significantly differentially expressed at a *P-*value < 0.05.

### CircRNA ORFs (cORFs) prediction

The prediction of cORFs was carried out as described previously [18]. We utilized the prediction algorithm (https://github.com/kadenerlab/cORF_pipeline) to predict the cORFs of predictive circRNAs. Briefly, each circRNA sequence was multiplied four times and the longest ORF spanning the circRNA junction was selected for each one of the three frames. All possible ORFs in a circRNA were scored by the following formula:


S=100*KNOWN\_ START+1000*HEAD\_ TO\_ TAIL+len(aa)/1,010,000*MOEBIUS_ORF

(1) KNOWN_START is 1 if the start codon coincides with the annotated start of the mRNA ORF. (2) HEAD_TO_TAIL is 1 if the cORF spans the head-to-tail junction. (3) MOEBIUS_ORF is 1 if the cORF has no stop codon and thus theoretically never terminates.

The cORF can be divided into COMPLETE, MOEBIUS, HEAD_TO_TAIL, and MICROPEPTIDE based on their attribute. (I) COMPLETE is meaning that all sequence in a circRNA is involved in the translation process; (Ⅱ) MOEBIUS is meaning that each circRNA sequence was multiplied four times and has no stop codon; (Ⅲ) HEAD_TO_TAIL is meaning that cORF spans the head-to-tail junction; (Ⅳ) MICROPEPTIDE is meaning that the protein length is less than 100 aa.

### Ribosome profiling of myoblasts

Chicken primary myoblasts at two time points (myoblasts achieving 50% cell confluence (designated GM group) and myoblasts differentiation for 3 days (designated DM group) were performed for Ribosome sequencing (Ribo-seq) following the original protocol [68] with minor modifications. Each interval included three biological replicates (**n* *= 3). Approximately 1 × 10^6^ cells were treated with cycloheximide (100 μg/mL) for 5 min. Next, the cells were lysed in lysis buffer (20 mM Tris–HCl, pH 7.4, 150 mM NaCl, 5 mM MgCl_2_, 1% Triton X-100, 1 mM DTT, 100 μg/mL cycloheximide, 20 U/ml TURBO DNase (Ambion, AM1907)) and incubated for 30 min on wet ice with occasional vortexing. Lysates were centrifuged at 20,000 × g for 10 min at 4°C. Then, the supernatant was collected and treated with 2 U/ml of RNase I (Ambion, AM2294) for 40 min at room temperature to digest RNA not protected by the ribosome. Digestion was stopped by the addition of 4.5 μL of SUPERaseIn RNase Inhibitor (Ambion, AM2696). Footprinted samples were pelleted through 1 M sucrose cushion by ultracentrifugation at 260,000 × g for 4 hours at 4°C. RNA pellets were resuspended in 10 mM Tris-HCl with 1% SDS (pH 7.4) and purified using RNA Clean & Concentrator-25 kit (Biozym). The isolated footprinted RNA was depleted of rRNA using the Illumina Ribo-Zero Gold kit (Illumina). The RNA was separated on a polyacrylamide gel to purify ribosome-protected fragments (RPF). Fragments (27–30 nt) of RNA were eluted from the gel and used for library construction with Truseq small RNA library kit (RS-200–0012 Illumina). Ribo-seq was sequenced on an Illumina NovaSeq with paired-end 150 bp reads. To minimize false positives when mapping ribosome footprints to back-splice junctions, we employed stringent criteria: (a) Ribosome-protected fragments (RPFs) were required to map uniquely and unambiguously across the back-splice junction with zero mismatches allowed within the junction-flanking regions. (b) A minimum threshold of ≥ 20 unique junction-spanning reads was imposed for circRNA RPF inclusion. (c) Putative circRNA-RPF mappings were cross-referenced against potential genomic rearrangements or annotated repetitive elements in the chicken genome (Gallus_gallus-5.0).

### Bioinformatic analysis of ribosome profiling data

The Ribo-seq raw reads were trimmed of the adaptor sequence by using cutadapt (v1.1) software [69]. Reads shorter than 20 nt were filtered, and low-quality reads were removed using PRINSEQ (v0.20.4) [70]. Reads with a length of 27–32 nt were kept because they are more likely to represent the ribosome-protected fragments. The reads were aligned to the ribosomal RNA database to remove rRNA reads using Bowtie 2 (v2.2.3). A layer alignment was performed to filter reads mapping to tRNA and snRNA sequences. The unaligned reads were then aligned to the chicken reference genome (ftp://ftp.ensembl.org/pub/release-73/fasta/gallus_gallus/dna/) using Tophat2 software. Ribosome profiles of individual genes were obtained by quantifying the coverage at a gene position by the 5′ ends of the reads.

### Small open reading frames (smORFs) encoded polypeptides (SEPs) extraction and enrichment

Chicken primary myoblasts (GM and DM group; three biological replications in each group; **n* *= 3) were performed for SEPs extraction and enrichment as described [68]. Briefly, approximately 4 × 10^7^ cells were lysed in lysis buffer (50 mM HCl, 0.1% *β*-mercaptoethanol (β-ME); 0.05% Triton X-100). After extraction, the extracts were centrifuged at 25,000 × g for 30 min at 4^o^C. Supernatants were collected and filtered through 5 μM syringe filters. The filtered solution was enriched for SEPs by using Bond Elute C8 silica cartridges (Agilent Technologies, Santa Clara, CA). Bond Elute C8 silica cartridges were first prepared with one column volume of methanol and equilibrated by using two-column volumes of triethylammonium formate (TEAF) buffer (pH = 3.0). After cellular extracts were applied, the cartridges were washed with two column volumes of TEAF, and the SEP enriched fraction was eluted by the addition of acetonitrile: TEAF = 3:1 (pH = 3.0). The enrichment SEP was lyophilized by a Savant Speed-Vac concentrator and the content of SEP was estimated using an enhanced BCA protein assay kit (Beyotime Biotechnology, China).

### Sample preparation and Nano-HPLC-MS/MS analysis

An aliquot of 100 μg of enriched samples was re-dissolved in 100 mM TEAB (pH = 8.5). Proteins were treated following reduction, alkylation, and trypsin digestion to obtain peptides according to the original protocol [71]. Nano-HPLC-MS/MS analysis was performed following a previous study [72]. Samples were resuspended with 30 μL solvent C (C: water with 0.1% formic acid), separated by nanoLC, and analyzed by online electrospray tandem mass spectrometry. The experiments were carried out by using a Nano Aquity UPLC system (Waters Corporation, Milford, MA, USA) coupled with a Q-Exactive plus mass spectrometer (Thermo Fisher Scientific, MA, USA) equipped with an online nano-electrospray ion source. A 10 μL peptide sample was loaded onto a trap column (Thermo Scientific Acclaim PepMap C18, 100 μm × 2 cm) with a flow of 10 μL/min for 3 min and then subsequently separated on the analytical column (Acclaim PepMap C18, 75 μm × 15 cm) with a 60 min linear gradient, from 5% D (D: ACN with 0.1% formic acid) to 55% D. The column was re-equilibrated at initial conditions for 10 min. The flow rate of the analytical column was maintained at 300 nL/min. The electrospray voltage of 2 kV versus the inlet of the mass spectrometer was used. The Q Exactive mass spectrometer was run under data-dependent acquisition mode to switch automatically between MS and MS/ MS acquisition. Survey full-scan MS spectra from 350 − 1,500 m/z were acquired with a mass resolution of 70 K, followed by 10 sequential high energy collisional dissociation (HCD) MS/MS scans with a resolution of 17.5 K. Dynamic exclusion was used with one microscan and 10 s exclusion duration. The mass spectrometry proteomics data have been deposited to the ProteomeXchange Consortium (http://proteomecentral.proteomexchange.org) via the iProX partner repository [73] with the dataset identifier PXD021030. ‍

### Protein data analysis to identify annotated and non-annotated SEPs

Tandem mass spectra were extracted from raw files using the Proteome Discoverer software (Thermo Fisher Scientific, version 1.4.0.288) and Nano-HPLC-MS/MS results were searched using MASCOT software (Matrix Science, London, UK; version 2.3).

The protein data analysis pipeline was carried out as described previously [16]. Two protein databases were utilized in these searches, a customized database containing peptides encoded by RNA sequences spanning back-splice junctions of all known circRNA and a combined UNIPROT Chicken database (Gallus gallus). To identify annotated and nonannotated SEP, nano-HPLC-MS/MS protein data files from biological replications (**n* *= 3) were combined and searched by using MASCOT. Data were searched with a precursor ion tolerance of 20 ppm and fragment mass tolerance of 0.7 Da. Proteins and SEP required at least one peptide to be identified with false discovery rates (FDR) lower than 1%. The peptides obtained from nano-HPLC-MS/MS across back-splice junction were filtered against the UNIPROT chicken database using a string-searching algorithm to ensure these peptides are not from any known chicken protein.

To determine whether the nonannotated peptides are from smORF, the nonannotated peptides are searched against NCBI Chicken Reference Sequence Database (RefSeq) by using tBLASTn. After identifying an RNA and sequence that encodes the peptide, we annotated it to upstream in-frame start codons and downstream in-frame stop codons. For the upstream in-frame start codon search, we firstly assigned start codons to any in-frame ATG. If there was no in-frame ATG, we searched for an in-frame near-cognate codon including GUG, ACG, CUG, AGG, AAG, AUU, and AUC, with a Kozak sequence in front of it. If neither an in-frame ATG nor a near-cognate start codon was identified, we searched the upstream in-frame stop codon. When the distance between the upstream and downstream in-frame stop codons was less than 150 codons, we annotated the gene as a smORF.

### RNase R treatment of total RNA

Total RNA from chicken myoblast was isolated using Trizol reagent (Invitrogen, Carlsbad, CA, USA) and treated with RNase R (Epicenter Technologies, Madison, WI). RNase R treatment was performed as follows: 3 mg of total RNA was digested with 5 U/μg RNase R, incubated for 15 min at 37°C, and purified by phenol-chloroform extraction.

### Validation of circRNA by Sanger sequencing

The validations of circRNA were performed by using PCR with divergent and convergent primers as described [74]. Firstly, obtained the full-length sequence, back-splice junction and exon information of circRNA from the sequencing results, and then designed the circRNA divergent primers crossing the back-splice junction, and convergent primers in regions of one exon. Both divergent and convergent primers were used in PCR with chicken muscle cDNA and genome DNA as template. To confirm the back-splice junction sequence of circRNA, PCR products of divergent primers were gel purified and submitted to Sangon Biotech Co. Ltd (Shanghai, China) for Sanger sequencing. All primers used for circRNA validation were listed in S13 Table.

### Complementary DNA (cDNA) synthesis and quantitative real-time PCR (qRT-PCR)

cDNA synthesis was carried out using a PrimeScript RT reagent Kit with gDNA Eraser (Perfect Real Time; Takara, Japan). The qRT-PCR reactions were performed in a QuantStudio5 Real-Time PCR Systems (Thermo Fisher, USA) with iTaq Universal SYBR Green Supermix Kit (Toyobo, Japan) along with four independent replications and three technical repetitions. The 2^-ΔΔCT^ method was used for the calculation of the relative RNA expression and all primers used for qRT-PCR were listed in S15 Table.

### Antibody generation and western blotting

The sequence of SIK2-176aa was cloned into the prokaryotic expression vector pET-28a (+). The recombinant vector was transformed into BL21 (DE3) and the expression of SIK2-176aa-his was induced by IPTG and purified using Ni-NTA Agarose (Qiagen, GER). Then a polyclonal antibody against the SIK2-176aa peptide produced by circSIK2 was obtained by inoculating rabbits and the antibody was purified using affinity chromatography columns. Cellular and tissue proteins were extracted by using radioimmunoprecipitation assay (RIPA) buffer with phenylmethanesulfonyl fluoride (PMSF) protease inhibitor. Western blotting was performed as previously described [75]. Simply, put samples underwent SDS-PAGE gel electrophoresis and subsequently were transferred to a PVDF membrane. The membrane was washed twice with TBST, and then blocked with a TBST solution containing 5% non-fat milk for 1 hour. Following this, the membrane was incubated with the primary antibody overnight at 4°C. Wash the membrane twice with TBST and incubate it for 1 hour with a suitable secondary antibody. Here, all the antibody information is provided as follows, and each protein band represents the number of replicates. The SIK2-176aa antibody was used at a 1:1,000 dilution. The other primary antibodies used for western blots were as follow: anti-flag antibody (Transgen Biotech, China), anti-CDKN1A antibody (bs0741R, Bioss, China), anti-CDKN1B antibody (bs0742R, Bioss, China), anti-CDKN2B antibody (bs4269R, Bioss, China), anti-Cyclin D2 antibody (AF5410, Affinity Biosciences, USA), anti-IGF1R antibody (bs0680R, Bioss, China), anti-INSR antibody (bs0681R, Bioss, China), anti-AKT1 (#9272, Cell Signaling Technology), anti-phospho-AKT1 (#4040, Cell Signaling Technology), anti-MyoD (ab16148, Abcam), anti-MyHC (B103, Developmental Studies Hybridoma Bank), anti-HNRNPA2B1 (Fab6995, Hunan Fenghui Biotechnology), anti-GAPDH (MB001, Bioworld Technology), anti-Tubulin (MB0009, Bioworld Technology). Goat anti-mouse IgG (H&L)-HRP (BS12478, Bioworld Technology) and goat anti-rabbit IgG (H&L)-HRP (BS13278, Bioworld Technology) were used as secondary antibodies.

### Plasmids construction, RNA oligonucleotides, and transfections

The circRNA overexpression vectors of circSIK2 were constructed using the linear sequences of circ_019191 amplified by PCR and subcloned into a commercially available circular RNA expression vector pCD25-ciR (Geneseed Biotech, China) using the *EcoRI* and *KpnI* restriction sites. We also synthesized a circSIK2-MT expression vector as a mutant control by changing the initiator codon ATG of circSIK2 into ACG at Sangon Biotech (Shanghai, China). The linear sequence of flag tagged SIK2-176aa, MYH9–167aa and CPSF6–102aa were cloned into pcDNA3.1 (+) overexpression vector. The linear sequence of flag-tagged RABGAPL-358aa and TRPC6–275aa were artificially synthesized by Sangon Biotech (Shanghai, China) and cloned into the pcDNA3.1 (+) vector. The full length of the METTL3 coding sequence (NCBI Reference Sequence: XM_040655036.2) was also synthesized by Sangon Biotech and cloned into the pcDNA3.1 (+) vector.

The potential methylation modification sites of circSIK2 were predicted through an online website: http://www.cuilab.cn/sramp [76]. Based on 7 potential m^6^A modification sites, circSIK2 fragments containing a different number of m^6^A sites were cloned into pmirGLO dual-luciferase reporter vector (Promega, USA) and named as pGLO-S1 (35–135, containing 131 m^6^A site), pGLO-S1-2 (78–184, containing 131 and 167 m^6^A sites), pGLO-S1-3 (78–207, containing 131, 167, and 202 m^6^A sites), pGLO-S1-4 (78–221, containing 131, 167, 202, and 213 m^6^A sites), pGLO-S1-5 (78–260, containing 131, 167, 202, 213, and 232 m^6^A sites), pGLO-S1-6 (78–382, containing 131, 167, 202, 213, 232, and 281 m^6^A sites), pGLO-S1-7 (78–446, containing 131, 167, 202, 213, 232, 281, and 400 m^6^A sites). The pGLO-S2 (151–200, containing 167 m^6^A site) was synthesized by Sangon Biotech (Shanghai, China) and cloned into the pmirGLO vector. These were numbered relative to the first base of the circSIK2.

The constructed plasmids were transfected with Lipofectamine 3000 (Invitrogen, Carlsbad, CA) following the manufacturer’s instructions. All primers used for plasmid construction were listed in S16 Table. The two target sequences of the siRNAs for circSIK2 were 5’ -TCAACACACCAAAGATTGT- 3’ (si1-circSIK2) and 5’ – CACCAAAGATTGTGAGCAT- 3’ (si2-circSIK2).

### Mass spectrometry for detection of circRNA-coded proteins

Cellular and tissue proteins were extracted using RIPA buffer and were separated via SDS-PAGE. The SDS-PAGE gel was visualized by Coomassie Brilliant Blue staining, and the target gel bands were manually cut and digested with sequencing-grade trypsin (Promega, Madison, WI). The digested peptides were analyzed with a Q-Exactive mass spectrometer (Thermo Fisher, Carlsbad, CA). The recorded spectra were analyzed using the Mascot (Matrix Science) using a custom-made database, with carbamidomethylating on cysteines as a fixed and oxidation of methionine’s considered as a static modification.

### Single-base elongation and ligation-based PCR amplification method (SELECT)

The SELECT method was used to verify the specific m^6^A-modified sites and the protocol was consistent with the previous description [77]. Briefly, specific reverse transcription primers for each m^6^A modified site and corresponding nonmodified site were designed for the following reverse transcription reaction (see S17 Table for SELECT primers). After digestion with RNase R, RNA (*n* = 3) was mixed with 40 nM primers, 5 µM dNTP, and 1.7 µL 10 × CutSmart buffer for a 17 µL total reaction solution and annealed as the following procedure: 90°C for 1 min, 80°C for 1 min, 70°C for 1 min, 60°C for 1 min, 50°C for 1 min, 40°C for 6 mins. Subsequently, add 3 uL of a mixture consisting of 0.01 U Bst 2.0 DNA polymerase, 0.5 U SplintR ligase, and 10 nM ATP into the first step reaction solution and run the single base ligation procedure: 40°C for 20 mins and 80°C for 20 mins. Finally, CT values were then compared using real-time PCR as described above.

### Dual-luciferase reporter assay

Each pmirGLO plasmid (100 ng) was co-transfected with METTL3 (50 ng) into DF-1 cells in 96 well plates with 8 replications. Firefly and Renilla luciferase activities were measured using a Dual-GLO Luciferase Assay System Kit (Promega, USA) after 48 h transfection in a Fluorescence/Multi-Detection Microplate Reader (BioTek, USA).

### F2-RNA pulldown assay

The F2-Tag is a short RNA sequence with a total of 16 nt (GGCGCTGACAAAGCGC) which hardly affects the structure or function of the target RNA. We divided the F2-Tag into two halves and added it to the head and tail of the circSIK2 sequence and cloned it into a pCD25-ciR vector so that the F2-Tag could only be detected when circRNA was generated. The circSIK2 RNA pulldown assay was performed by using an F2-RNA pulldown assay kit (Fitgene, China) according to the manufacturer’s instructions after 48 h transfection of 14 µg F2-Tag plasmid in 10 cm cell plate. The eluted products were identified by mass spectrometry with a Q-Exactive Plus mass spectrometer or western blot.

### EdU (5-ethynyl-2’-deoxyuridine) assay

After 48h transfection of 1 µg of the plasmid or 100 nM siRNA in 12-well cell plates, chicken primary myoblasts were incubated with 50μM 5-ethynyl-2’-deoxyuridine (EdU; RiboBio) for 2h and then stained with a C10310 EdU Apollo In Vitro Imaging Kit (RiboBio, China). Leica DMi8 fluorescent microscope was used here to capture the EdU-stained or Hoechst 33342-stained cells with 4 wells and 6 randomly selected fields.

### Flow cytometric analysis

The chicken primary myoblasts were collected in 70% ethanol at −20°C overnight after a 48h transfection of 1 µg of the plasmid or 100 nM siRNA in 12-well cell plates with 4 wells replications. Then the cells were treated with a Cell Cycle Analysis Kit (Thermo Fisher Scientific, USA), and the BD AccuriC6 flow cytometer (BD Biosciences, USA) and FlowJo7.6 software was used for the flow cytometric analysis.

### Cell counting kit-8 (CCK-8) assay

After being transfected with 0.1 µg of the plasmid or 100 nM siRNA in 96-well cell plates (n = 8), we monitored the cell proliferation every 12 h by using a TransDetect CCK (Transgen Biotech, China), and absorbance was measured at a wavelength of 450 nm in a Model 680 Microplate Reader (Bio-Rad, USA).

### Immunofluorescence

The transfected chicken primary myoblasts (4 wells each group) were induced to differentiate for 3 days, after which the resulting myotubes were first treated with 4% formaldehyde and then with 0.1% Triton X-100. After blocking with goat serum, cells were then incubated with anti-MyHC (DHSB, USA) overnight and Goat Anti-Mouse IgG (H + L)-Dylight 594 (BS10027, Bioworld) for 1h after that. DAPI (Beyotime, China) was used for the nuclei staining. ImageJ software (National Institutes of Health) was used to count the myotube area.

### Lentiviral intramuscular injections and HE staining

The SIK2-176aa lentiviral vector was constructed, produced, and infected by GenePharma (China) using the LV5 (EF-1a/GFP&Puro) vector. Lentivirus was injected three times into the pectoral muscles of 1-day-old chicks (**n* *= 10) at days 1, 4, and 7 at a dosage of 1 × 10^8^ IU/mL. The pectoral muscles were obtained 14 days after the first injection and fixed in 10% formalin. Then the fixed tissues were paraffin-embedded, sectioned, and stained with hematoxylin and eosin. Over 500 muscle fibers were counted and analyzed per sample (*n* = 10) in ECHO Pro software, and the results were presented as mean ± S.E.M.

### Statistical analysis

All experimental results are presented as mean ± S.E.M with at least three independent replications. The statistically significant difference between groups was tested by independent sample t-test. We considered p < 0.05 in a comparison between groups to be statistically significant. *p < 0.05; **p < 0.01; ***p < 0.001.

## Supporting information

S1 Fig Annotation of circRNA in chicken myoblast and myotube. (A) Distribution of sequencing reads of identified circRNA. x-axis: the back-spliced read numbers of circRNA identified by circRNA-seq. y-axis: the abundance of circRNA classified by different read numbers. (B) Length distribution of the sequenced circRNA. x-axis: the sequence length distribution of detected circRNA. y–axis: the abundance of circRNA classified by different lengths. (C) Distribution of identified circRNA in chicken genome. The bar graphs illustrate the location of the detected circRNA within different chromosomes in GM (green bars) and DM (orange bars) groups, respectively. (D) The number of circRNAs distributed in different chromosomes. The bar graphs illustrate the numbers of circRNAs within different chromosome identified in GM (green bars) and DM (orange bars) group, respectively.
(DOCX)

S2 Fig The characteristic distinction between circRNA and their host mRNA. (A) Full-length distribution of circRNA and host mRNA. The full length of circRNA was shorter than their host mRNA, on average. (B) Exon number distribution for circRNA and host mRNA. The number of exons in host mRNA was more than circRNA. (C) CDS length distribution of circRNA and host mRNA. CircRNA shares similar characteristics with host mRNA in the length of CDS. (D) UTR length distribution of circRNA and host mRNA. The UTR length of host mRNA was slightly longer than circRNA.
(DOCX)

S3 Fig Conservativeness analysis of 10 encoding circRNAs. (DOCX)

S4 Fig qRT-PCR detection of the effects of linear vectors on circRNA and proliferation, and differentiation related genes. (DOCX)

S5 Fig METTL3 could promote the expression of SIK2-176aa in DF-1 cells. (DOCX)

S1 Table RNA-seq was performed to identify circRNA in chicken myoblasts (GM) and myotubes (DM). (XLSX)

S2 Table The original information of circRNA-seq in each sample. (XLSX)

S3 Table Full-length distribution of circRNA and host mRNAs. (XLSX)

S4 Table Exon number distribution for circRNA and host mRNAs. (XLSX)

S5 Table CDs length distribution of circRNA and host mRNAs. (XLSX)

S6 Table UTR length distribution of circRNA and host mRNAs. (XLSX)

S7 Table List of circRNA cORF in chicken myoblast and myotube. (XLSX)

S8 Table List of circRNA cORF contains no stop codon. (XLSX)

S9 Table List of chicken ribo-circRNA. (XLSX)

S10 Table List of chicken ribo-circRNA contains IRES elements. (XLSX)

S11 Table List of circRNA without any stop codon associated with ribosome. (XLSX)

S12 Table List of the combination of peptides encoded by all circRNAs and the UNIPROT Chicken database (Gallus gallus). (XLSX)

S13 Table List of all primers used for circRNA validation. (XLSX)

S14 Table List of all circSIK2 specific binding proteins by RNA pulldown coupled with mass spectrometry. (XLSX)

S15 Table List of all primers used for qRT-PCR. (XLSX)

S16 Table List of all primers used for plasmid construction. (XLSX)

S17 Table List of all SELECT primers. (XLSX)
