# Supplementary material for: METTL3 facilitates the translation of CircSIK2 during chicken myogenesis in an m6A dependent manner
Source: PLoS Genet. 2025 Oct 31;21(10):e1011934. doi: 10.1371/journal.pgen.1011934 (PMC12578262; doi:10.1371/journal.pgen.1011934)
Supplement: S1 Fig — (A) Distribution of sequencing reads of identified circRNA. x-axis: the back-spliced read numbers of circRNA identified by circRNA-seq. y-axis: the abundance of circRNA classified by different read numbers. (B) Length distribution of the sequenced circRNA. x-axis: the sequence length distribution of detected circRNA. y–axis: the abundance of circRNA classified by different lengths. (C) Distribution of identified circRNA in chicken genome. The bar graphs illustrate the location of the detected circRNA within different chromosomes in GM (green bars) and DM (orange bars) groups, respectively. (D) The number of circRNAs distributed in different chromosomes. The bar graphs illustrate the numbers of circRNAs within different chromosome identified in GM (green bars) and DM (orange bars) group, respectively. (DOCX) [file pgen.1011934.s001.docx]

Supplementary Figures

S1 Fig: Annotation of circRNA in chicken myoblast and myotube.

(**A**) Distribution of sequencing reads of identified circRNA. x-axis: the back-spliced read numbers of circRNA identified by circRNA-seq. y-axis: the abundance of circRNA classified by different read numbers. **(B)** Length distribution of the sequenced circRNA. x-axis: the sequence length distribution of detected circRNA. y–axis: the abundance of circRNA classified by different lengths. (**C**) Distribution of identified circRNA in chicken genome. The bar graphs illustrate the location of the detected circRNA within different chromosomes in GM (**green bars**) and DM (**orange bars**) groups, respectively. (**D**) The number of circRNAs distributed in different chromosomes. The bar graphs illustrate the numbers of circRNAs within different chromosome identified in GM (green bars) and DM (orange bars) group, respectively.
