# Supplementary material for: METTL3 facilitates the translation of CircSIK2 during chicken myogenesis in an m6A dependent manner
Source: PLoS Genet. 2025 Oct 31;21(10):e1011934. doi: 10.1371/journal.pgen.1011934 (PMC12578262; doi:10.1371/journal.pgen.1011934)
Supplement: S2 Fig — (A) Full-length distribution of circRNA and host mRNA. The full length of circRNA was shorter than their host mRNA, on average. (B) Exon number distribution for circRNA and host mRNA. The number of exons in host mRNA was more than circRNA. (C) CDS length distribution of circRNA and host mRNA. CircRNA shares similar characteristics with host mRNA in the length of CDS. (D) UTR length distribution of circRNA and host mRNA. The UTR length of host mRNA was slightly longer than circRNA. (DOCX) [file pgen.1011934.s002.docx]

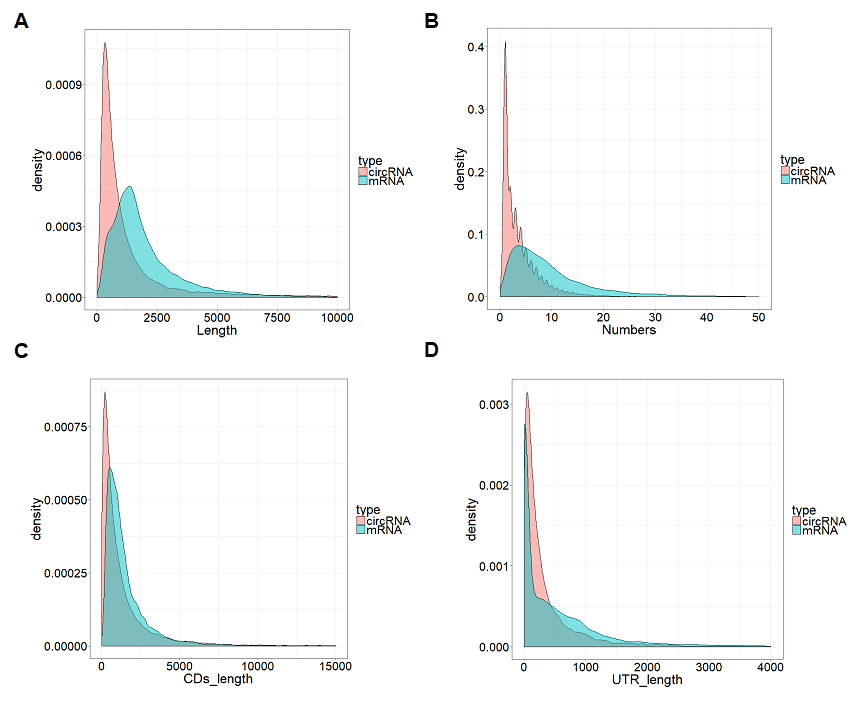


S2 Fig: The characteristic distinction between circRNA and their host mRNA.

(A) Full-length distribution of circRNA and host mRNA. The full length of circRNA was shorter than their host mRNA, on average. (B) Exon number distribution for circRNA and host mRNA. The number of exons in host mRNA was more than circRNA. (C) CDS length distribution of circRNA and host mRNA. CircRNA shares similar characteristics with host mRNA in the length of CDS. (D) UTR length distribution of circRNA and host mRNA. The UTR length of host mRNA was slightly longer than circRNA.
