# Supplementary figures and images for: METTL3 facilitates the translation of CircSIK2 during chicken myogenesis in an m6A dependent manner
Source: PLoS Genet. 2025 Oct 31;21(10):e1011934. doi: 10.1371/journal.pgen.1011934 (PMC12578262; doi:10.1371/journal.pgen.1011934)

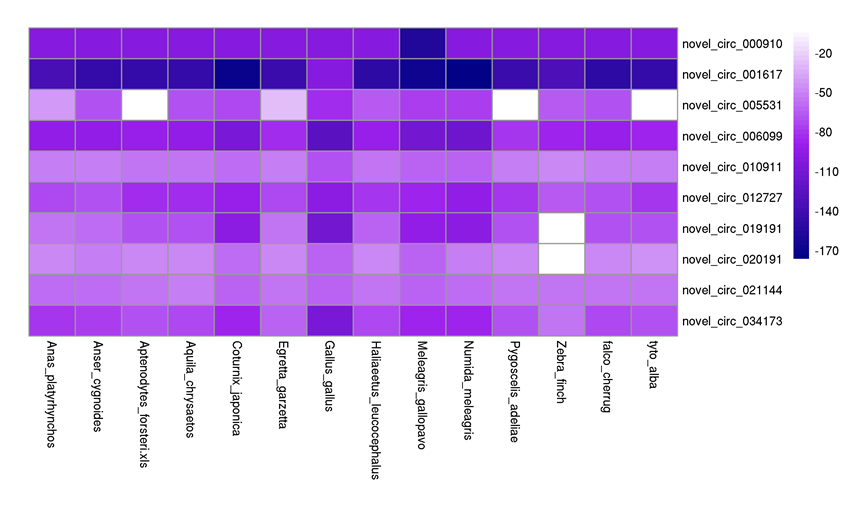


S3 Fig: Conservativeness analysis of 10 encoding circRNAs.

Supplement: S3 Fig — (DOCX) [file pgen.1011934.s003.docx]

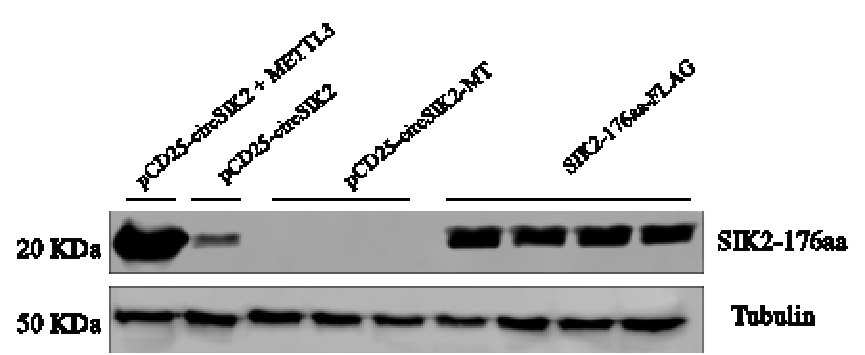


S5 Fig: METTL3 could promote the expression of SIK2-176aa in DF-1 cells

Supplement: S5 Fig — (DOCX) [file pgen.1011934.s005.docx]
