# Supplementary material for: METTL3 facilitates the translation of CircSIK2 during chicken myogenesis in an m6A dependent manner
Source: PLoS Genet. 2025 Oct 31;21(10):e1011934. doi: 10.1371/journal.pgen.1011934 (PMC12578262; doi:10.1371/journal.pgen.1011934)
Supplement: S4 Fig — (DOCX) [file pgen.1011934.s004.docx]

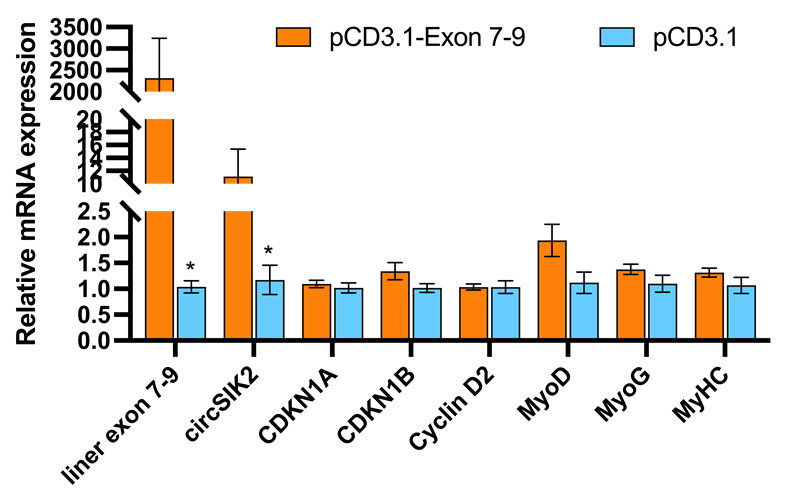


S4 Fig: qRT-PCR detection of the effects of linear vectors on circRNA and proliferation, and differentiation related genes
